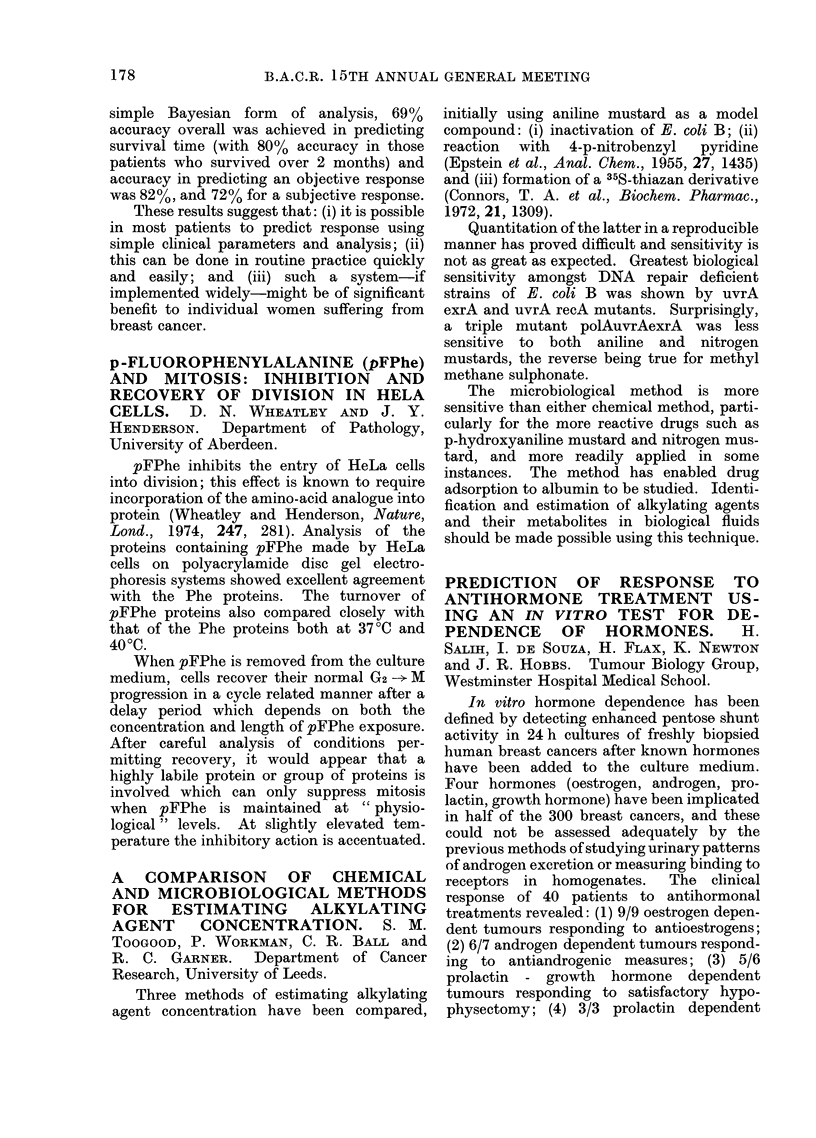# Proceedings: Para-Fluorophenylalanine (pFhe) and mitosis: inhibition and recovery of division in HeLa cells.

**DOI:** 10.1038/bjc.1974.148

**Published:** 1974-08

**Authors:** D. N. Wheatley, J. Y. Henderson


**p-FLUOROPHENYLALANINE (*p*FPhe) AND MITOSIS: INHIBITION AND RECOVERY OF DIVISION IN HELA CELLS.** D. N. WHEATLEY AND J. Y. HENDERSON. Department of Pathology, University of Aberdeen.

*p*FPhe inhibits the entry of HeLa cells into division; this effect is known to require incorporation of the amino-acid analogue into protein (Wheatley and Henderson, *Nature, Lond.*, 1974, **247**, 281). Analysis of the proteins containing *p*FPhe made by HeLa cells on polyacrylamide disc gel electrophoresis systems showed excellent agreement with the Phe proteins. The turnover of *p*FPhe proteins also compared closely with that of the Phe proteins both at 37°C and 40°C.

When *p*FPhe is removed from the culture medium, cells recover their normal $G_2 \rightarrow M$ progression in a cycle related manner after a delay period which depends on both the concentration and length of *p*FPhe exposure. After careful analysis of conditions permitting recovery, it would appear that a highly labile protein or group of proteins is involved which can only suppress mitosis when *p*FPhe is maintained at " physiological " levels. At slightly elevated temperature the inhibitory action is accentuated.